# PROGmiR: a tool for identifying prognostic miRNA biomarkers in multiple cancers using publicly available data

**DOI:** 10.1186/2043-9113-2-23

**Published:** 2012-12-28

**Authors:** Chirayu Pankaj Goswami, Harikrishna Nakshatri

**Affiliations:** 1 Center for Computational Biology and Bioinformatics, Indiana University School of Medicine, 410 W 10th Street, Indianapolis, IN, 46202, USA; 2 Departments of Surgery, Biochemistry and Molecular Biology, Indiana University School of Medicine, Indianapolis, IN, 46202, USA

**Keywords:** miRNA, Prognostics, Cancer, Pan-cancer, Database, Signature, Biomaker

## Abstract

**Background:**

Identification of prognostic biomarkers is hallmark of cancer genomics. Since miRNAs regulate expression of multiple genes, they act as potent biomarkers in several cancers. Identification of miRNAs that are prognostically important has been done sporadically, but no resource is available till date that allows users to study prognostics of miRNAs of interest, utilizing the wealth of available data, in major cancer types.

**Description:**

In this paper, we present a web based tool that allows users to study prognostic properties of miRNAs in several cancer types, using publicly available data. We have compiled data from Gene Expression Omnibus (GEO), and recently developed “The Cancer Genome Atlas (TCGA)”, to create this tool. The tool is called “PROGmiR” and it is available at http://www.compbio.iupui.edu/progmir. Currently, our tool can be used to study overall survival implications for approximately 1050 human miRNAs in 16 major cancer types.

**Conclusions:**

We believe this resource, as a hypothesis generation tool, will be helpful for researchers to link miRNA expression with cancer outcome and to design mechanistic studies. We studied performance of our tool using identified miRNA biomarkers from published studies. The prognostic plots created using our tool for specific miRNAs in specific cancer types corroborated with the findings in the studies.

## Background

MiRNAs are small non coding RNA molecules, 18-25 bases at maturity, and exert a profound effect on regulation of genetic machinery of the cell. MiRNAs are found in both plant and animal cells and act as key regulators of gene expression. The first miRNA, a small transcript of lin-4 gene, was discovered as an antisense molecule to lin-14 mRNA in C. elegans [1]. Lin-14 gene synthesizes lin-14 protein, lower levels of which are essential for normal embryonic development of C. elegans larvae. The first miRNA to be categorically studied was 21 nucleotide long let-7 miRNA [2]. MiRNA were subsequently also discovered in plants [3]. MiRNA are found in almost every species ranging from unicellular organisms such as yeast to primates, but the number of miRNAs in different species ranges from a few dozens to thousands.

Since miRNAs act as key regulators of gene expression, their role is evident in several diseases where gene expression is altered. For this reason, despite their recent discovery, miRNAs have been studied to a great extent in cancers, where pathological changes are bought about primarily due to altered gene expression. Recent studies have suggested a role of miRNAs in many cancers. MiRNAs have so far been reported to be up or down regulated in several types of cancers [4]. In cancer, miRNAs may act as tumor suppressors or tumor promoters (oncogenes). MiR17-92 cluster is a prominent oncogenic miRNA cluster [5], whereas miR-34 family, which is induced by the tumor suppressor gene p53 [6], displays tumor suppressor activity in multiple cancers. MiRNAs have been reported to play these roles in both cancers of hematopoietic origin as well as in solid tumors. Also, several miRNAs have been found to be directly associated with tumor progression and metastasis [7-10].

The first miRNA to be associated with cancer was discovered in Chronic Lymphocytic Leukemia (CLL). Croce et al discovered a novel miRNA signature associated with prognosis and disease progression in CLL [11]. By systematic profiling of miRNAs in several cancers, Golub et al [12] observed a general downregulation of miRNAs in cancer samples. Subsequently, miRNAs were discovered in several cancers to be of prognostic importance. For instance, Monzo et al recently discovered a 25 miRNA signature for Hodgkin’s Lymphoma (HL) and also identified miR-135a as a key player in prognostic outcome of HL [13]. In breast cancer, miR-155 [14] and miR-21 [15] are upregulated, while miR-10b has been implicated in invasiveness and metastasis of breast cancer [7]. MiR17-5p, miR-20a, miR-335, and miR-126 along with other miRNAs have also been found to be of importance in breast cancer [8,16]. In two separate studies, miR-143 and miR-145 were found to be downregulated in colorectal cancer [17,18]. Murakami et al analyzed Hepatocellular Carcinoma (HCC), chronic hepatitis and normal liver tissue for miRNAs and found an eight miRNA signature predictive of HCC [19]. MiR-224, miR-18, and pre-mir-18 were upregulated and miR-199a*, miR-200a, miR-199a, miR-125a, and miR-195 were downregulated in HCC samples compared to normal liver samples. In recent studies, miR-21, miR-10b, and miR-222 were found to be upregulated and miR-200c and miR-203 were downregulated in HCC. In Non Small Cell Lung Cancer (NSCLC), miR-128b functions as a tumor suppressor miRNA by controlling the production of EGFR [20]. MiR-128b along with a five miRNA signature constituting of miR-221, miR-137, miR-372, miR-182, and let-7 has been shown to be predictive of outcome in NSCLC patients [21]. In pancreatic cancer, separate studies identified miR-126 as a biomarker for pancreatic cancer [22-24].

MiRNA regulate gene expression in several ways, especially in pathological situations. Altered expression of some genes, particularly transcription factors, may result in altered expression of other miRNAs, which as a feed forward action causes altered expression of yet other genes resulting in heavy deregulation of normal molecular machinery of the cell. For example, we reported recently specific down-regulation of miR-22 in metastatic breast cancer cells compared to primary tumor cells, which results in elevated expression of the transcription factor EVI-1 in metastatic cancer cells [25]. EVI-1 being an epigenetic modulator of gene expression can profoundly alter gene expression pattern in metastatic cells.

Altered expression of miRNAs in cancers may occur due to genetic abnormalities, altered transcription, altered post-transcriptional events, or altered epigenetic factors. Consequently, dysregulated miRNAs alter cellular machinery at genomic/epigenomic level. For instance, miR-29 inhibits the expression of DNMT3A and DNMT3B, which are involved in DNA methylation in lung cancer [26], whereas miR-101 has been shown to regulate histone methyltransferase EZH2 in prostate cancer [27]. MiR-15a and miR-16-1 were one of the first miRNAs to be discovered whose expression was altered due to genetic abnormality in chronic lymphocytic leukemia [28]. In a global analysis of mouse genome, several miRNAs were found to be associated with sites of frequent genomic abnormalities [29]. A similar trend was observed in the human genome while studying cancers [30]. Altered transcriptional regulation of miRNAs is also a major cause of deregulation of miRNA expression in certain cancers. It has been noted that transcription factors (TFs) may induce miRNAs by activating transcription of pri-miRNAs. Several miRNAs have also been experimentally shown to be directly regulated by TFs in cancers and quite often these microRNAs target the same transcription factors that induce them as a feedback loop [31-39]. miRNA expression can also be altered during post transcriptional events. For instance, levels of miRNA processing enzymes DROSHA and DICER were found to be altered in many cancers [40-44]. Also, in silico analysis of breast cancer tissue specimens has identified CpG islands near dozens of miRNAs genomic locations [45]. Furthermore, DNA hypomethylation induced release of miRNA silencing in colorectal cancer [46] suggests an epigenetic regulation of miRNA expression in cancers.

Altered miRNA expression can bring about patho-physiological changes in cellular machinery in several ways. Important mechanisms are induction of apoptosis, alteration of cell cycle, increased invasive and metastatic characteristics in the cells. For example, miR-29b [47], miR-34s [31], miR-15a and miR-16 [48] participate in tumorigenesis by targeting anti-apoptotic genes. MiR-221 and miR-222 in Glioblastoma (GBM) [49] and prostate cancer [50] have been shown to target p27, which restricts the cell cycle to G1 stage by preventing G1-to-S transition. As mentioned before, miR-10b [6] is suggested to impart metastatic characteristics to the cell, while miR-18 and miR-19 have been reported to repress TSP-1 and CTGF [37], both control angiogenesis.

All the above mentioned studies emphasize the remarkable multifaceted role miRNAs play as potent biomarkers in cancer. The biomarker capabilities of miRNAs have been studied sporadically (in specific studies for specific cancers), and the data for many such studies are available in public repositories. Global profiling of miRNAs in cancers has been done using the traditional PCR techniques, but more frequently using array platforms and sequencing. Recent advances in sequencing technologies have enhanced our capacity to study the global transcriptome of cancer populations, including miRNAs and other non-coding RNAs. Currently, there is no resource available which enables users to study biomarker capabilities of miRNAs in different cancers. Researchers have to go through searching and processing publically available datasets for identifying and published information on prognostic implications of miRNA in cancer of interest. The wealth of data available publically thus remains underutilized, and this motivated us to create a platform where miRNAs can be studied for several types of cancers as prognostic biomarkers. In this paper, we present a first of a kind web based tool for studying prognostic importance of miRNAs in several types of cancers. Our tool is called PROGmiR, and it is available online freely for academic and non commercial purposes. PROGmiR allows users to study overall survival in form of prognostic plots using miRNA expression data from several publically available patient series. The data in our tool comes from Gene Expression Omnibus (GEO) and The Cancer Genome Atlas (TCGA) [51]. We have compiled the data on 16 different cancer types from both these sources. A list of cancer types available in our database along with other statistics on the data sets is available in Table 1. Our tool uses miRNA expression data from these datasets to create overall survival Kaplan-Meier (K-M) plots. Plots can be created for individual miRNAs as well as for average expression of a group (signature) of miRNAs in any of the cancer mentioned in Table 1.

**Table 1:** Description of various data sources included in progmir database

	**Cancer type**	**Dataset**	**Source**	**Platform**	**No of samples**	No of miRNAs*	**Reference**
1	Adrenocortial Carcinoma	GSE22816	GEO	Agilent-025987 Human miRNA Microarray Release 14.0	22	200	[52]
2	Acute Myeloid Leukemia	LAML	TCGA	Illumina GA miRNA Seq	164	704	[51]
3	Brain Lower Grade Glioma	LGG	TCGA	Illumina HISeq miRNA Seq	29	1046	[51]
4	Glioblastoma multiforme	GBM	TCGA	UNC miRNA 8X15K	487	470	[51]
5	Breast Invasive Carcinoma	BRCA	TCGA	Illumina GA and HISeq	727	1046	[51]
6	Non-small-cell Lung Cancer	GSE16025	GEO	mirVANA miRNA Bioarray V2	60	328	[53]
7	Small Cell Lung Cancer	GSE27435	GEO	Capitalbio mammal microRNA V3.0	42	1638	[54]
8	Lung Adenocarcinoma	LUAD	TCGA	Illumina GA and HISeq	79	1046	[51]
9	Lung Squamous Cell Carcinoma	LUSC	TCGA	Illumina GA and HISeq	185	1046	[51]
10	Hepatocellular Carcinoma	GSE31384	GEO	CapitalBio custom Human microRNA array	166	682	[55]
11	Head and Neck Squamous Cell Carcinoma	HNSC	TCGA	Illumina GA and HISeq	89	1046	[51]
12	Ovarian Cystadenocarcinoma	OV	TCGA	UNC miRNA 8X15K	46	705	[51]
13	Rectal Adenocarcinoma	READ	TCGA	Illumina GA miRNA Seq	38	705	[51]
14	Renal Clear Cell Carcinoma	KIRC	TCGA	Illumina GA and HISeq	546	1046	[51]
15	Stomach Ademocarcinoma	STAD	TCGA	Illumina GA and HISeq	79	1046	[51]
16	Uterine Corpus Endometroid Carcinoma	UCEC	TCGA	Illumina GA and HISeq	358	1046	[51]

Currently, our tool is restricted to studying only overall survival, but in the future, as more data become publically available, we aim to extend our tool to enable users to study additional prognostic measures such as metastasis free survival, recurrence free survival, and effects of specific therapies using our tool. Currently we present this tool as a hypothesis generation tool only. In future versions, we would also like to add demographic and clinical covariates such as age, race and hormonal and therapy statuses to be included in the analysis to produce more meaningful and accurate prognostic plots. Since miRNAs have significant importance as biomarkers, and since our tool covers almost all major cancers from more than 10 tissues of origin, we believe this tool will help researchers in identifying novel prognostic markers as well as in formulating hypothesis for mechanistic studies in the future. In particular, our tool will allow investigators to obtain preliminary evidence on whether microRNA functions as a tumor suppressor or oncogene across all cancer types or has dual role depending on cancer type.

## Construction and content

### Workflow

**Figure 1:**
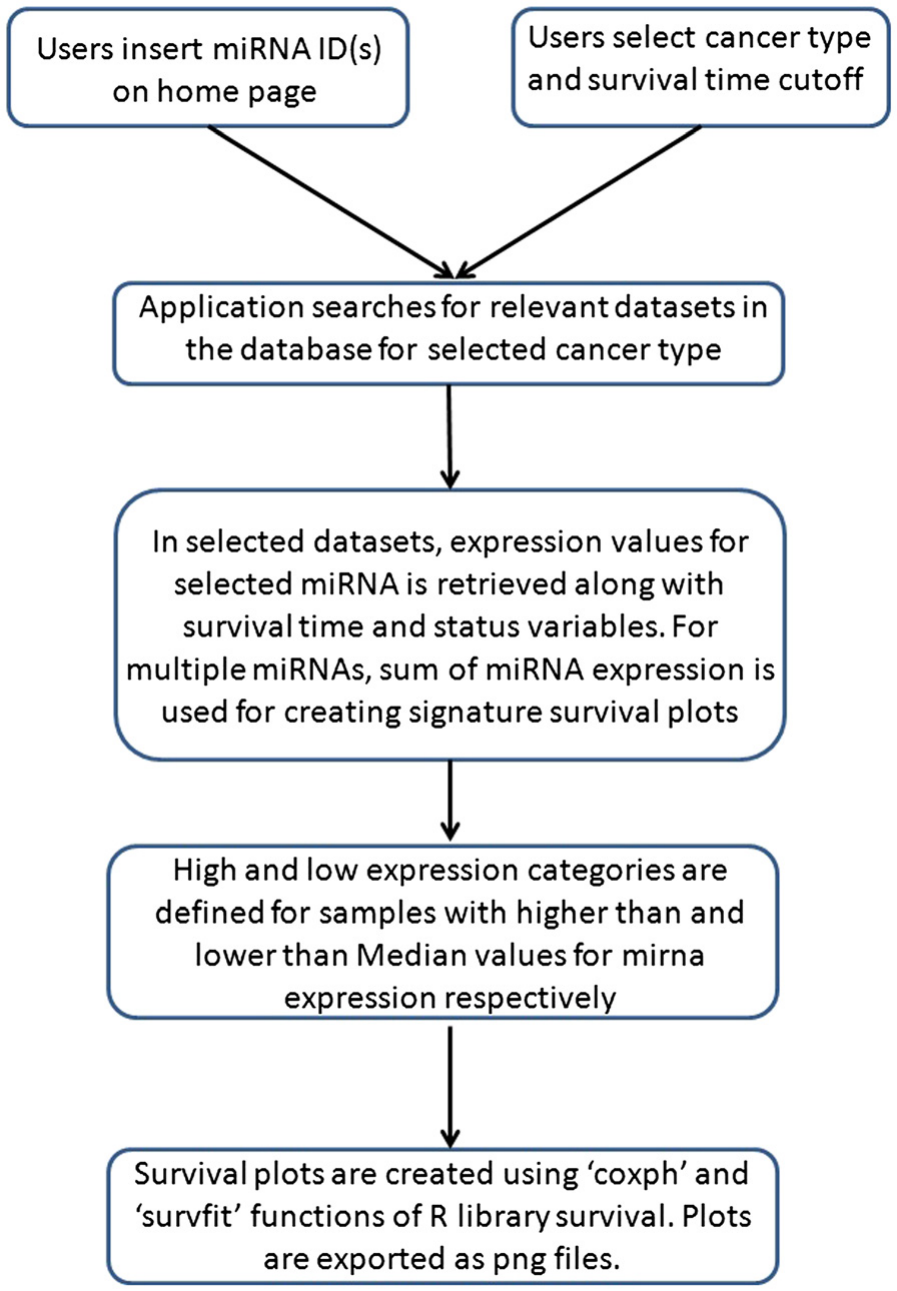
Line diagram delineating workflow of PROGmiR.

To create prognostic plots for miRNAs, our application uses miRNA expression and overall survival data. MiRNA expression data are in form of array expression data or sequencing data based on the platform. Our application is a web based PHP [56] script which uses R [57] in the backend to compute survival plots for miRNAs of interest. The R script uses library ‘Survival’ for creating survival plots. Survival plots can be created for 3-year, 5-year or full follow up survival time. Figure 1 describes the workflow of the application. Prognostic plots in our application can be created for single or multiple miRNAs. When multiple miRNAs are entered, our application creates prognostic plots for miRNAs individually as well as a combined prognostic plot for all miRNAs entered. This makes it possible to study prognostic implications of a miRNA signature in different types of cancer. For combined plots, sum of miRNA expressions of all miRNAs entered is computed and used in creation of prognostic plots.

Users enter miRNA ID(s) on the home page of web application and select the cancer type in which prognostic plots have to be visualized. For some cancer types, there are more than one expression dataset present. For each dataset, one prognostic plot is created for each miRNA and one plot is created for sum of expressions of all miRNAs entered combined. The program retrieves expression data for each entered miRNA along with corresponding survival annotations. Hazard ratio and *p* value are then calculated using function ‘coxph’ and prognostic plots are visualized using function ‘survfit’, both functions in library ‘survival’ for R. To estimate hazard ratio and corresponding *p* value, continuous miRNA expression is used. For creating prognostic plots, samples are categorized into ‘HIGH’ and ‘LOW’ miRNA expression categories by bifurcating at median miRNA expression. The plots are exported as .png files and these files are visualized on the results page as individual miRNA plots and sum of expression plots divided by datasets. For each plot, ‘exp(Coef)’ as estimate of hazard ratio, and Log likelihood *p* value generated by the ‘coxph’ model are also provided.

### Data

We downloaded miRNA expression data from GEO and TCGA. GEO data were downloaded for separate platforms as described in Table 1. The TCGA data were in form of RNA sequencing data except for Glioblastoma, where it was on array platform. Sequencing data from TCGA were available in form of ‘reads per million (Level 3)’ for each miRNA. GEO data were downloaded in form of series matrices from the GEO website. Survival data associated with clinical samples were also downloaded from the respective data sources along with the expression data. The time to death data were converted into days to death for data sources where months or years to death were reported. Batch effect arising due to processing of samples at different times was removed statistically, if present. In most cases, unlike mRNA expression data, the data available were in already processed format and did not have to be normalized further. Since we do not combine expression data from all datasets for a particular cancer type, any other normalization was again not relevant. The data were stored as R data sets and computation is done directly using these datasets in our application.

### Web application

We have created a web application for implementation of our tool. As mentioned previously, the web application was written in PHP5 and uses R scripts in backend to create survival plots. The web application consists of a home page and results page. Users can input individual miRNAs or a comma delimited list of miRNAs on the home page. Users also select one cancer type in which prognostic plots have to be created. Also available on the home page is options to truncate plots to a 3 Yr or 5 Yr follow up or to create plots for full follow up time. Since different studies have been performed on different platforms, the number of miRNAs profiled in each study differs. Prognostic plots are created for each dataset for available miRNAs only. Upon submitting the information on home page, results in form of KM plots are provided on the results page.

## Utility and discussion

We have compiled miRNA expression data from 16 cancer types in our data base. Median survival and follow-up times as well as number of events for each dataset have been summarized in Table 2. We used only overall survival data in our database, as other survival functions such as metastasis free survival and relapse free survival, were not available for all studies. Studies in which numbers of survival events were less than 5 were not included in the database. In TCGA data, there is an innate problem of several samples been annotated as having a zero follow-up time. Such samples were removed from the final datasets. Our final data consists of a total of 3117 samples in 16 cancer types and appximately 1050 miRNAs profiled. Unlike related applications available for studying survival implications of mRNAs in breast and ovarian cancers [58,59], we did not merge all samples for the same cancer type into one large dataset owing to the fact that it may lead to over-fitting of data due to normalization of samples coming from different studies. For this reason, we kept different datasets belonging to the same cancer type separate.

**Table 2:** Global statistics on survival related variables for the datasets available in PROGmiR

				**Survival event**		**No event**	
	**Cancer type**	**Dataset**	**Total no of samples**	**No of samples**	**Median survival**^**§ **^**(range)**	**No of samples**	**Median survival**^§* ^**(range)**
1	Adrenocortial Carcinoma	GSE22816	22	6	600 (120-4860)	16	885 (60-4980)
2	Acute Myeloid Leukemia	LAML	164	100	303 (28-1706)	64	699 (28-2861)
3	Brain Lower Grade Glioma	LGG	29	23	788 (96-1915)	6	1317 (242-6423)
4	Glioblastoma multiforme	GBM	487	383	377 (3-3880)	104	266 (3-2817)
5	Breast Invasive Carcinoma	BRCA	727	91	1563 (157-4456)	636	466 (1-6795)
6	Non-small-cell Lung Cancer	GSE16025	60	26	1980 (210-2490)	34	1500 (120-2580)
7	Small Cell Lung Cancer	GSE27435	42	18	743 (234-2400)	24	1314 (426 -2490)
8	Lung Adenocarcinoma	LUAD	79	27	701 (22-1318)	52	400 (1-2161)
9	Lung Squamous Cell Carcinoma	LUSC	185	77	544 (12-5296)	108	640 (3-4299)
10	Hepatocellular Carcinoma	GSE31384	166	73	450 (30 – 2280)	93	1320 (420-2430)
11	Head and Neck Squamous Cell Carcinoma	HNSC	89	27	395 (128 – 2318)	62	359 (45 – 4115)
12	Ovarian Cystadenocarcinoma	OV	46	21	887 (9-1756)	25	912 (141 – 2099)
13	Rectal Adenocarcinoma	READ	38	3	316 (59 – 1184)	35	183 (28 – 2192)
14	Renal Clear Cell Carcinoma	KIRC	546	173	722 (2-2830)	373	1307 (4 – 3377)
15	Stomach Adenocarcinoma	STAD	79	14	258 (19 – 881)	65	109 (2 – 2131)
16	Uterine Corpus Endometroid Carcinoma	UCEC	358	27	413 (58 – 3251)	331	527 (1 – 5690)

§ in days.

* Follow up time.

**Figure 2:**
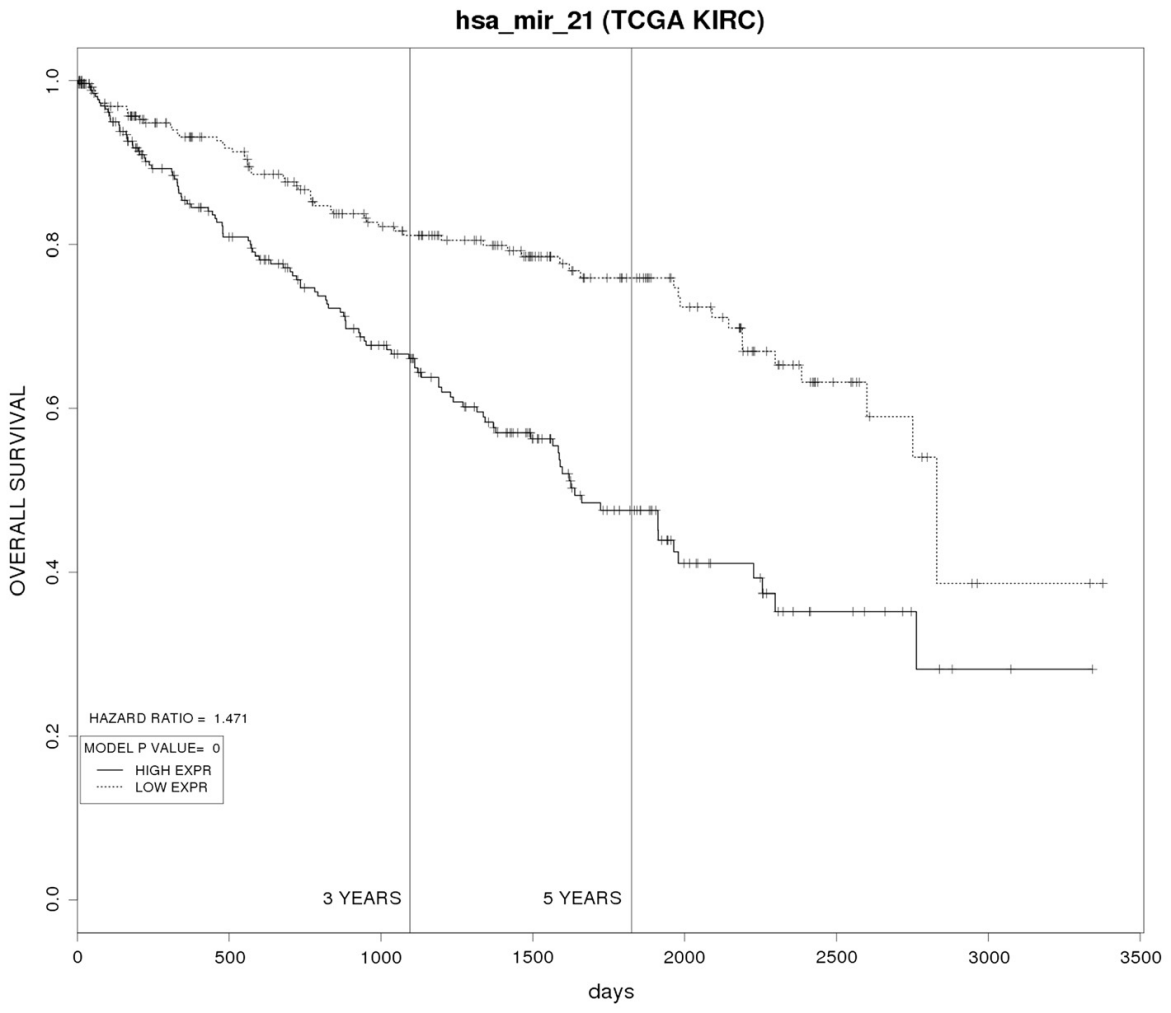
Prognostic plot created using PROGmiR for miR-21 identified as prognostic biomarker in Renal Clear Cell Carcinoma using TCGA data.

For assessing performance of our tool, we created survival plots for miRNAs that have been implicated as having prognostic importance in published studies. Dahiya et al [60], have shown that a high expression of miR-21 correlates with poor overall survival in renal clear cell carcinoma. We used our tool to create prognostic plot for miR-21 in renal cell carcinoma in TCGA data. Figure 2 shows a poor overall survival for patients with high miR-21 expression compared to patients having low miR-21 expression levels. The hazard ratio and *p* value for the proportional hazards model is also given in the figure.

In another study, Chen et al [61], have demonstrated down-regulation of several isoforms of miR-181 (miR-181-a, miR-181-b, miR-181-c and miR-181-d) being correlated with poor overall survival in acute myeloid leukemia (AML). We used our tool to create prognostic plots for the above mentioned miRNA isoforms using TCGA data. All the isoforms and combined sum of expression of isoforms showed the same pattern in plots created using our tool as shown in the paper, with poor overall survival observed in the group having low expression of miR-181 isoforms. Additional file 1: Figures S1-S4 provided in supplementary data show prognostic plots for miR-181 isoforms in AML data. Additional file 1: Figure S5 shows a prognostic plot for sum of the 4 miRNAs showing correlation of overall survival with sum of miRNA expression. Similarly, in another study, Annilo et al [62], have demonstrated low expression of miR-374a to be associated with poor outcome in non small cell lung cancer. We used our tool to create overall survival prognostic plots for miR-374a in squamous cell carcinoma of lung (LUSC) using TCGA data. The plot (Additional file 1: Figure S6) showed the miRNA low expression arm demonstrating a poor outcome in LUSC samples.

In each of the case studies we performed, prognostic plots created by our tool corroborated with the findings in the published studies. Since this tool is basically a pipeline, this fact makes the tool validated. Somasundaram et al [63] recently used the TCGA data itself to identify 10 miRNAs having prognostic implications in glioblastoma. Our tool used the same data and showed similar results (Additional file 1: Figures S7-S16) for the selected 10 miRNAs.

In future updates to our tool, we plan to expand the repository of our tool by adding new datasets as and when they become available. We also plan to include more survival functions, such as ‘metastasis free survival’ and ‘relapse free survival’ to our tool in future versions.

## Conclusions

We believe this tool will prove useful for hypothesis generation and testing as well as for mechanistic studies. Considering the impact of miRNAs as prognostic biomarkers in several cancer types, such preliminary findings will also benefit researchers formulate research plans. Since our tool covers data from all major cancer types, researchers working on wide array of cancers will be able to formulate their study hypotheses based on findings from this tool.

## Availability and requirements

The database is available freely for non commercial and academic usage at http://www.compbio.iupui.edu/progmir.

## Competing interest

Both authors hereby declare no competing interests.

## Authors’ contributions

CG carried out study planning, data procurement, data management, quality assurance, data analysis, programming and web development, and manuscript preparation for the article. HN contributed to study planning and manuscript preparation. Both authors read and approved the final manuscript.

## Grant support

This study was partly supported by IUPUI Breast Cancer Signature Center and Susan G. Komen for the Cure grant SAC110025 (to HN).

## Supplementary Material

Additional file 1 **Figures S1-S4.** Prognostic plot created using PROGmiR for isoforms a, b, c and d of miRNA hsa-miR-181 identified as prognostically important biomarker in Acute Myeloid Leukemia (AML) by Chen et al, using TCGA data. **Figure S5**. Prognostic plot for sum of expression of hsa-mir-181 isoforms a,b,c and d in TCGA AML data. **Figure S6**. Prognostic plot created using PROGmiR for miRNA hsa-miR-374 identified as prognostically important biomarker in Lung Squamous cell carcinoma (LUSC) by Annilo et al, using TCGA data. **Figures S7-S16**. Prognostic plot created using PROGmiR for 10 miRNAs identified as prognostically important biomarker in Glioblastoma (GBM) by Somasundaram et al, using TCGA data. Click here for file
