# Supplementary material for: PROGmiR: a tool for identifying prognostic miRNA biomarkers in multiple cancers using publicly available data
Source: J Clin Bioinforma. 2012 Dec 28;2:23. doi: 10.1186/2043-9113-2-23 (PMC3564827; doi:10.1186/2043-9113-2-23)
Supplement: Additional file 1 — Figures S1-S4. Prognostic plot created using PROGmiR for isoforms a, b, c and d of miRNA hsa-miR-181 identified as prognostically important biomarker in Acute Myeloid Leukemia (AML) by Chen et al, using TCGA data. Figure S5. Prognostic plot for sum of expression of hsa-mir-181 isoforms a,b,c and d in TCGA AML data. Figure S6. Prognostic plot created using PROGmiR for miRNA hsa-miR-374 identified as prognostically important biomarker in Lung Squamous cell carcinoma (LUSC) by Annilo et al, using TCGA data. Figures S7-S16. Prognostic plot created using PROGmiR for 10 miRNAs identified as prognostically important biomarker in Glioblastoma (GBM) by Somasundaram et al, using TCGA data. [file 2043-9113-2-23-S1.pdf]

Supplemental Data for manuscript entitled

# PROGmiR: A tool for identifying prognostic miRNA biomarkers in multiple cancers using publicly available data.

Supplementary figures 1-4

Prognostic plot created using PROGmiR for isoforms a, b, c and d of miRNA hsa-miR-181 identified as prognostically important biomarker in Acute Myeloid Leukemia (AML) by Chen et al, using TCGA data

Figure 1

hsa-mir-181a-1

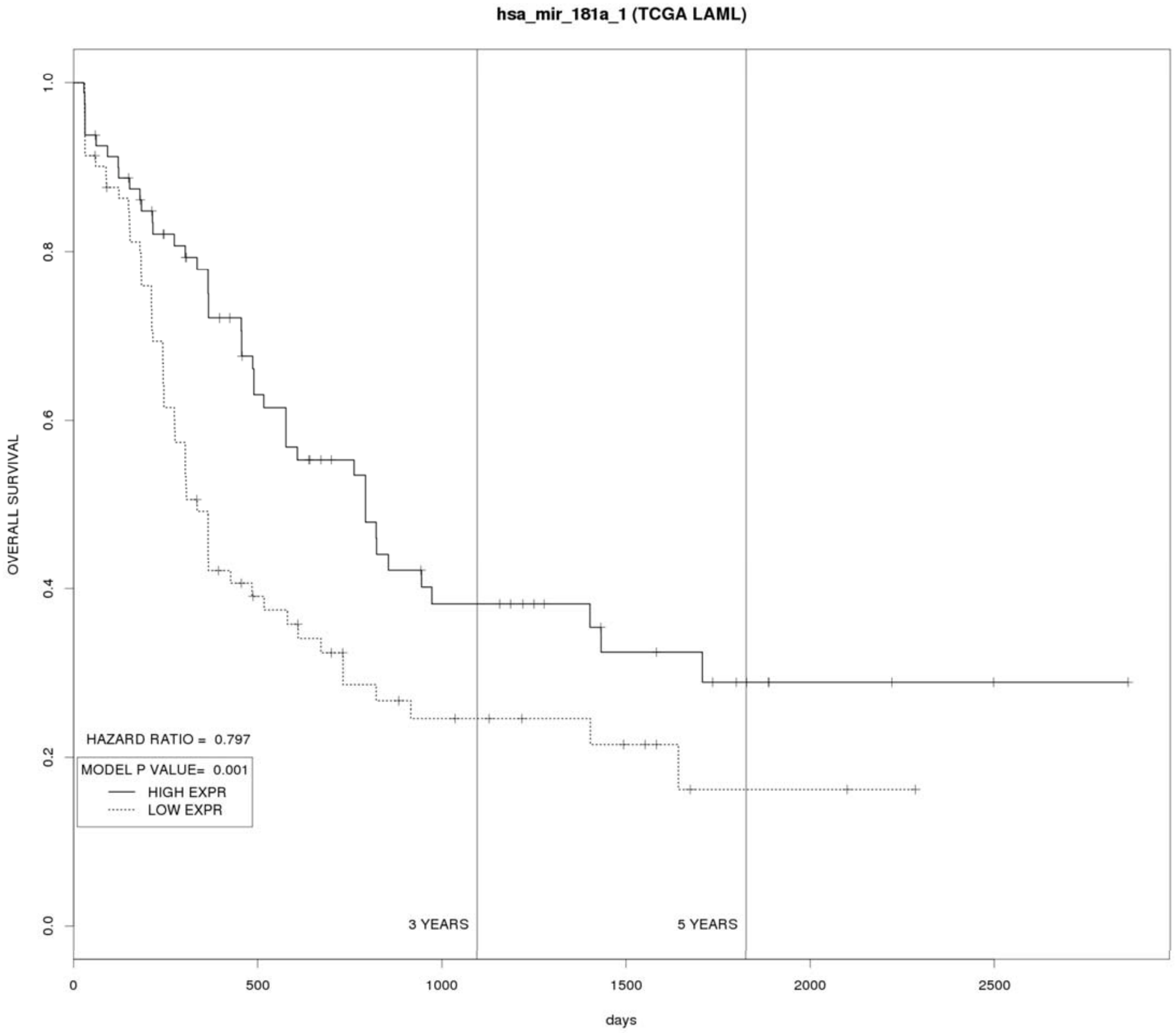

Figure 2

hsa-mir-181b-1

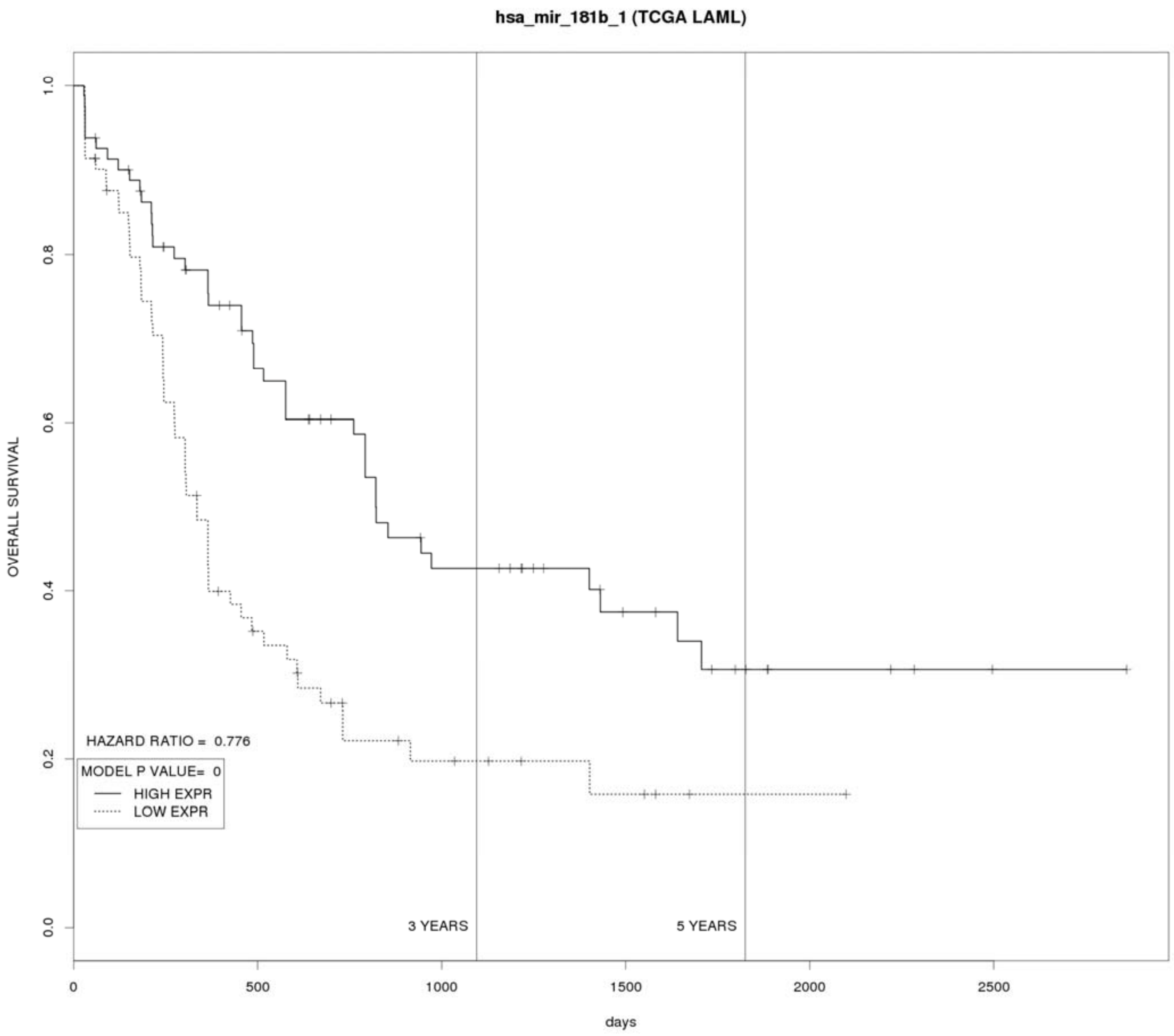

Figure 3

hsa-mir-181c

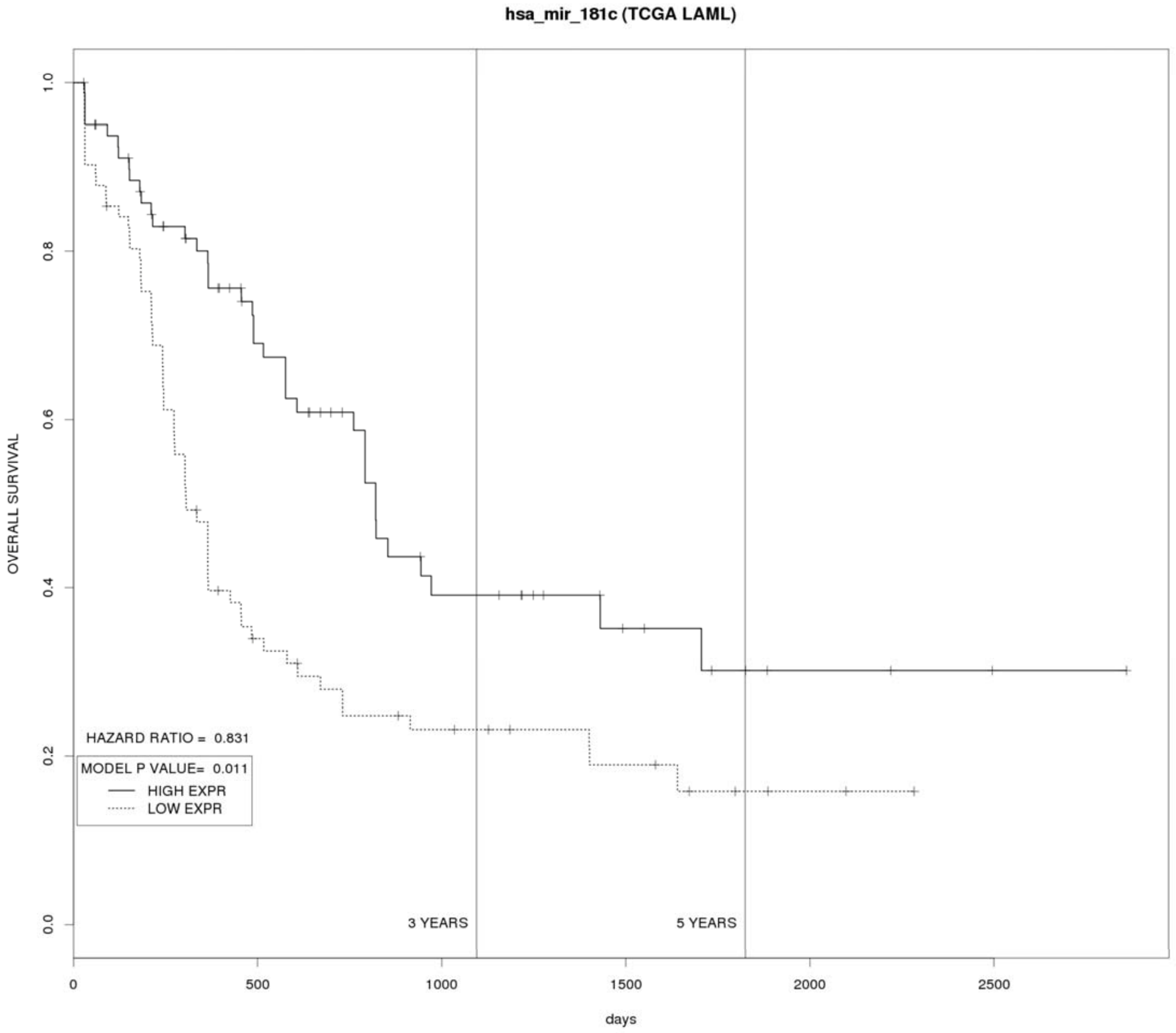

Figure 4

hsa-mir-181d

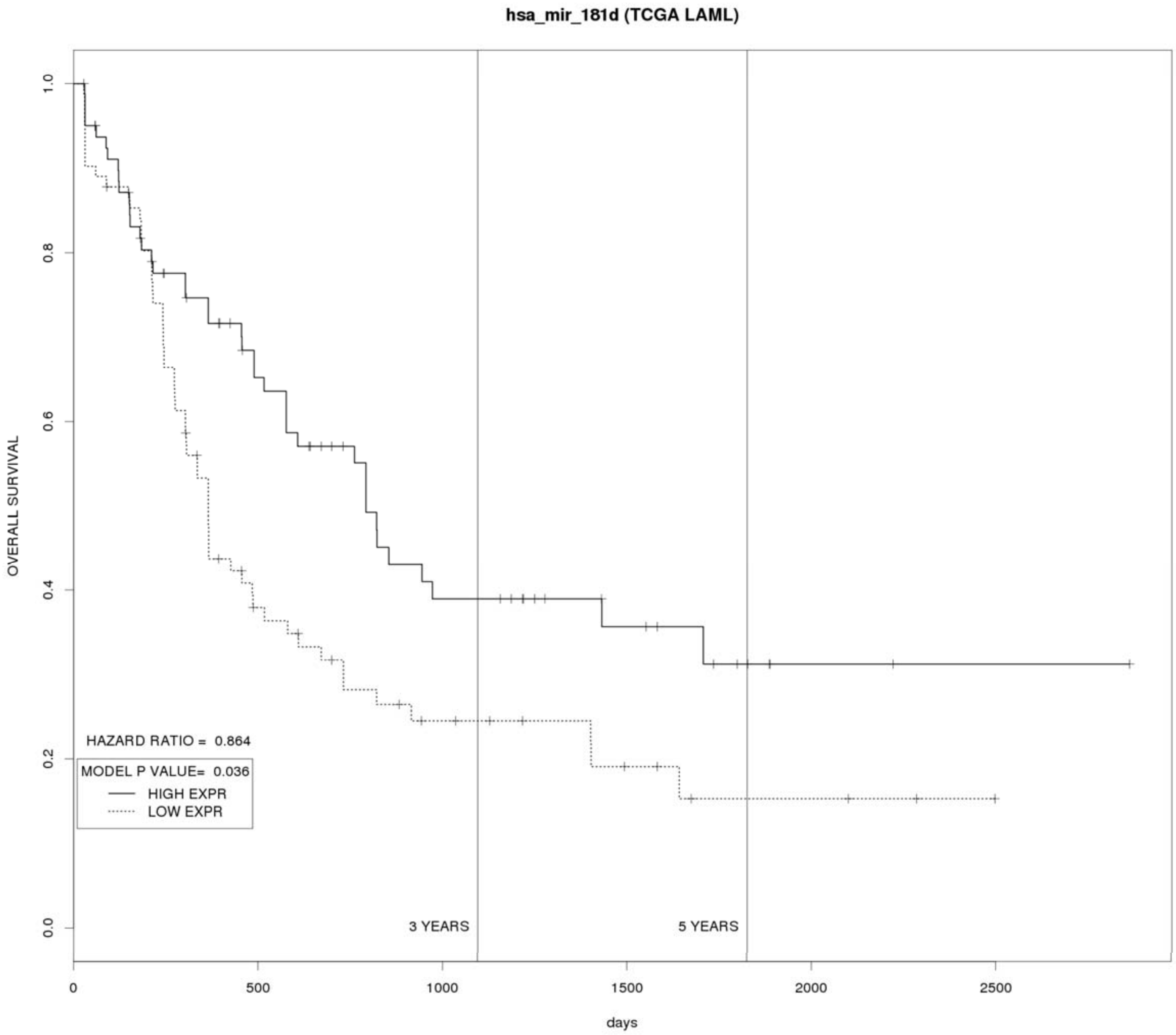

Figure 5

Prognostic plot for sum of expression of hsa-mir-181 isoforms a,b,c and d in TCGA AML data

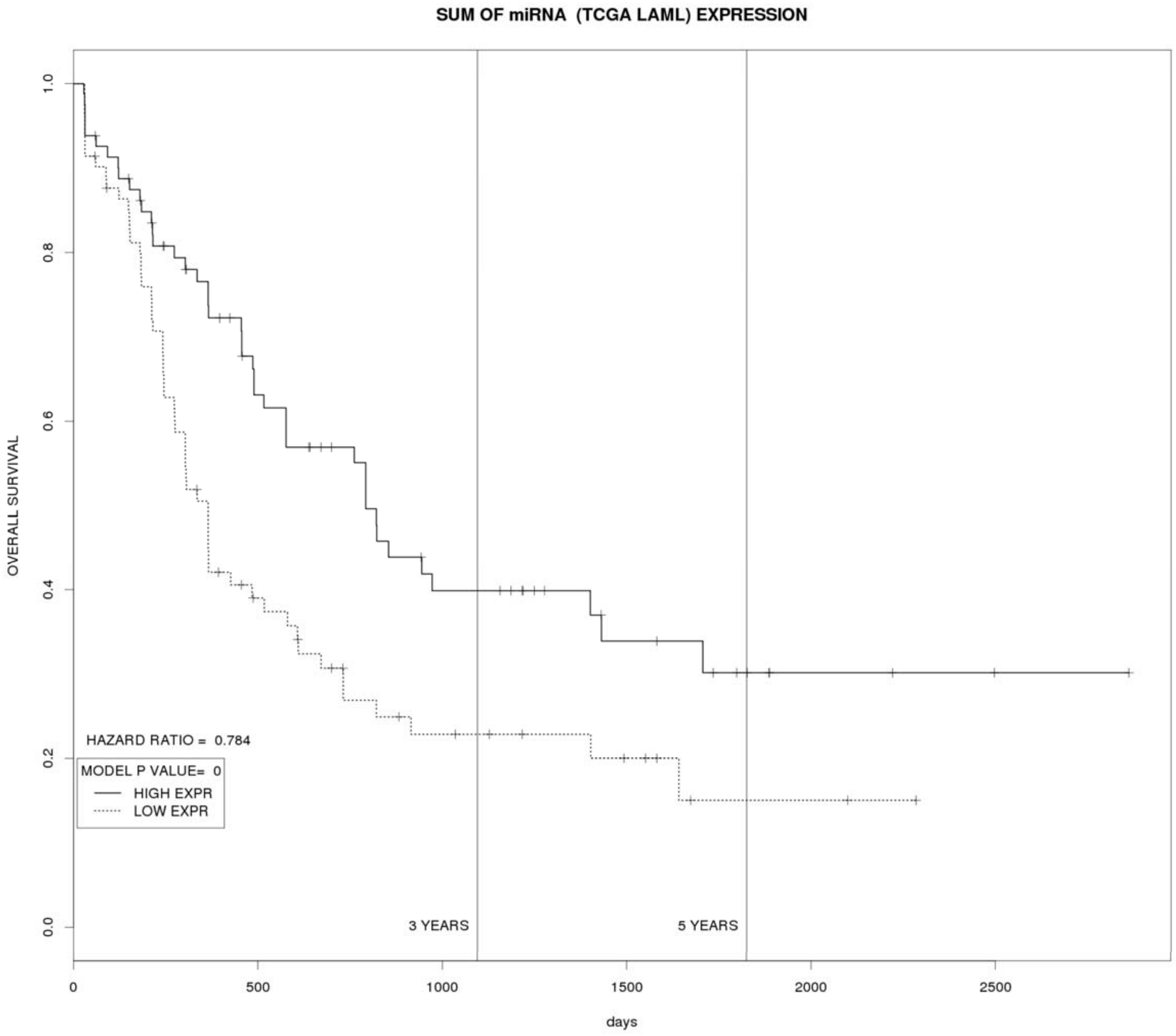

Figure 6

Prognostic plot created using PROGmiR for miRNA hsa-miR-374 identified as prognostically important biomarker in Lung Squamous cell carcinoma (LUSC) by Annilo et al, using TCGA data

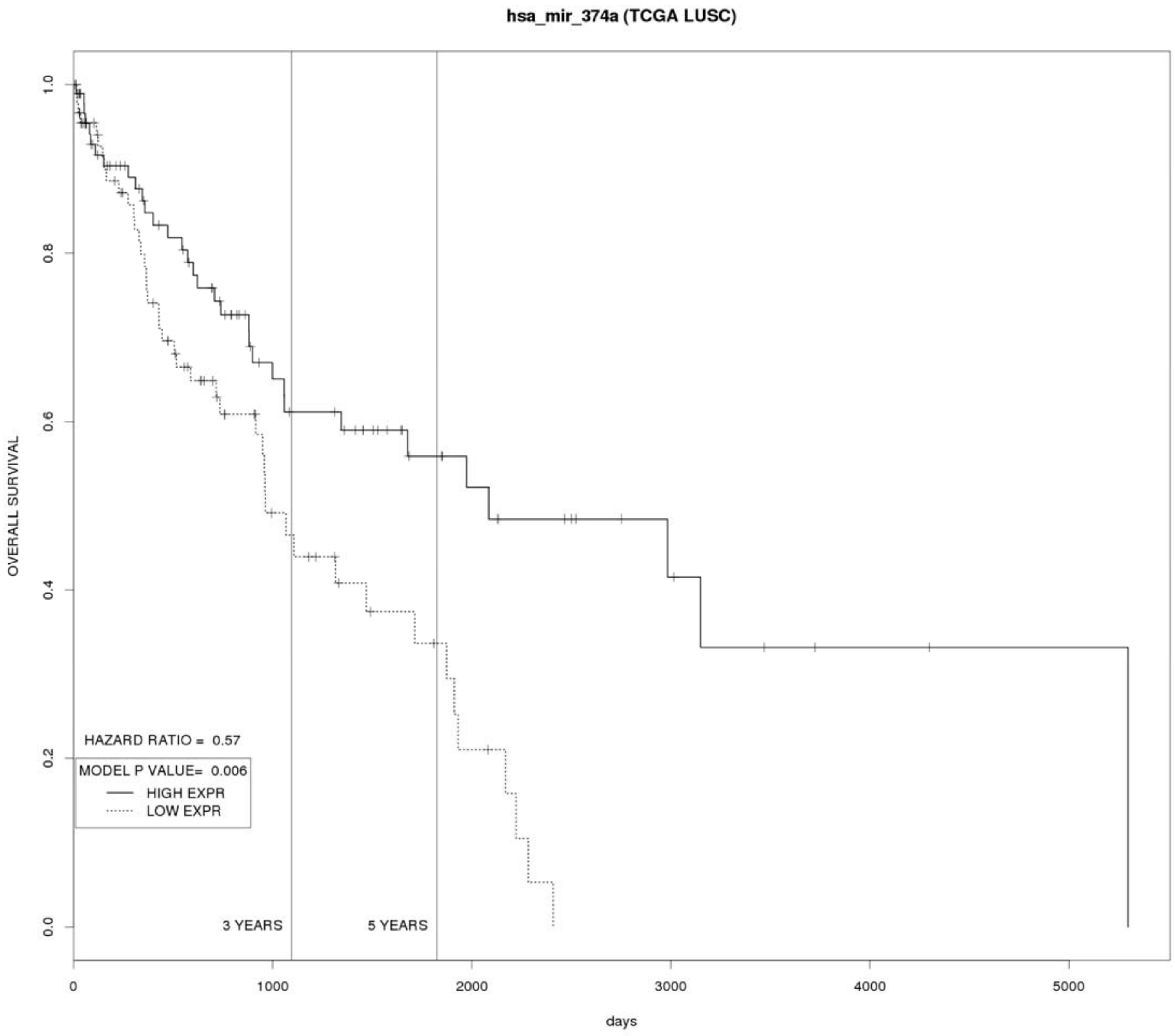

Supplementary figures 7-16

Prognostic plot created using PROGmiR for 10 miRNAs identified as prognostically important biomarker in Glioblastoma (GBM) by Somasundaram et al, using TCGA data

Figure 7

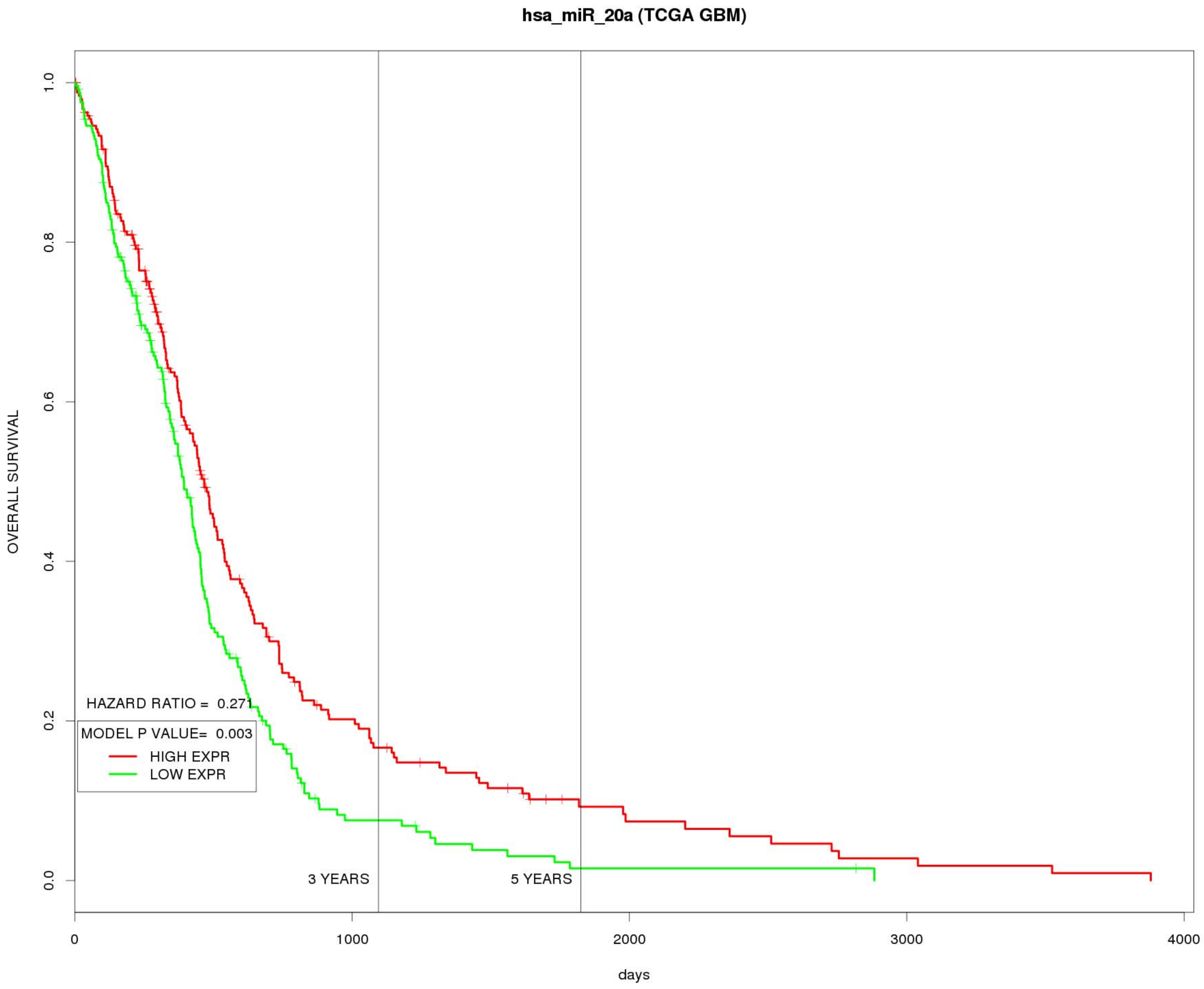

hsa_miR_20a (TCGA GBM)
OVERALL SURVIVAL
1.0
0.8
0.6
0.4
0.2
0.0
HAZARD RATIO = 0.271
MODEL P VALUE= 0.003
HIGH EXPR
LOW EXPR
3 YEARS
5 YEARS
0
1000
2000
3000
4000
days

Figure 8

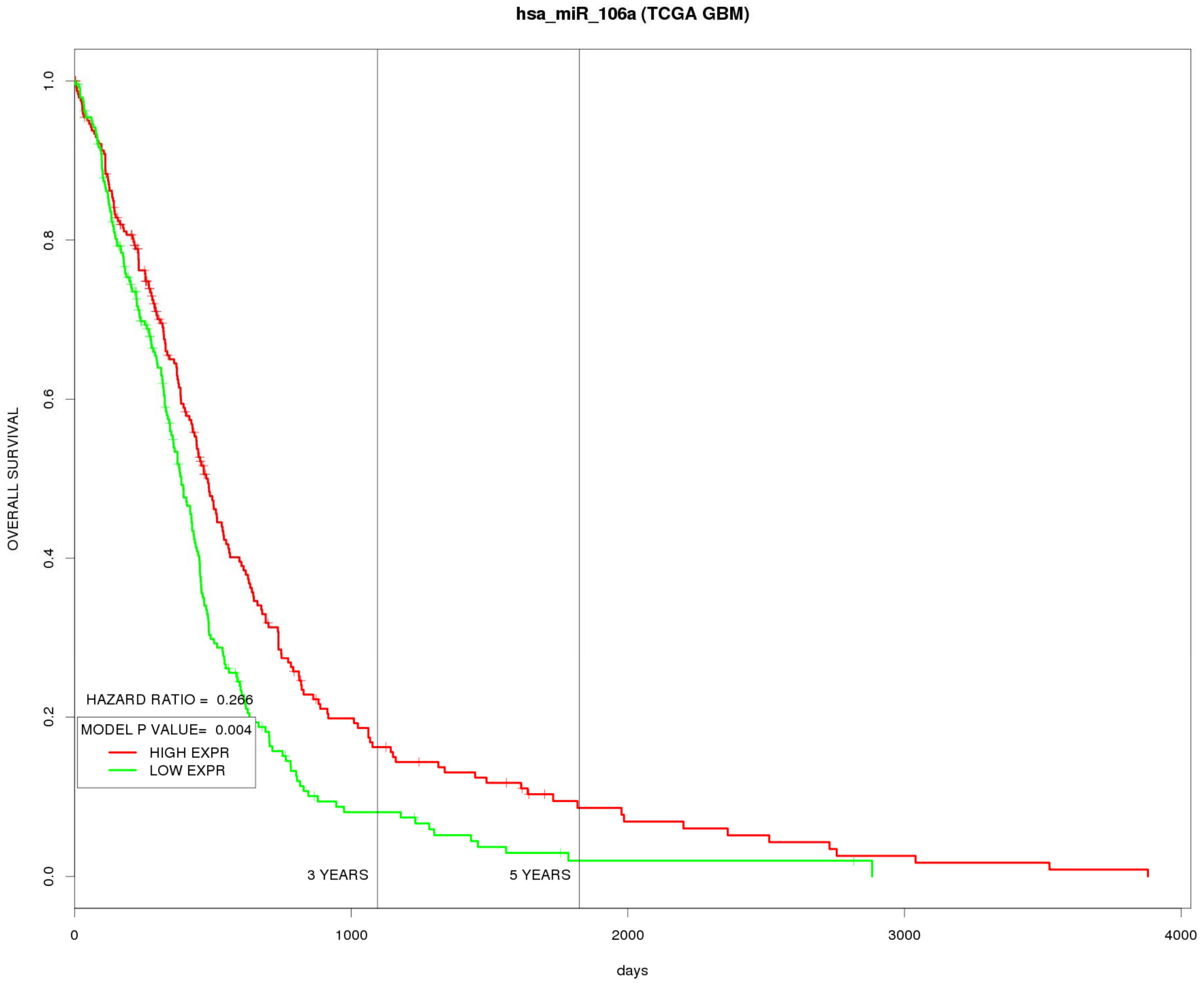

hsa_miR_106a (TCGA GBM)
OVERALL SURVIVAL
1.0
0.8
0.6
0.4
0.2
0.0
3 YEARS
5 YEARS
HAZARD RATIO = 0.266
MODEL P VALUE= 0.004
HIGH EXPR
LOW EXPR
0
1000
2000
3000
4000
days

Figure 9

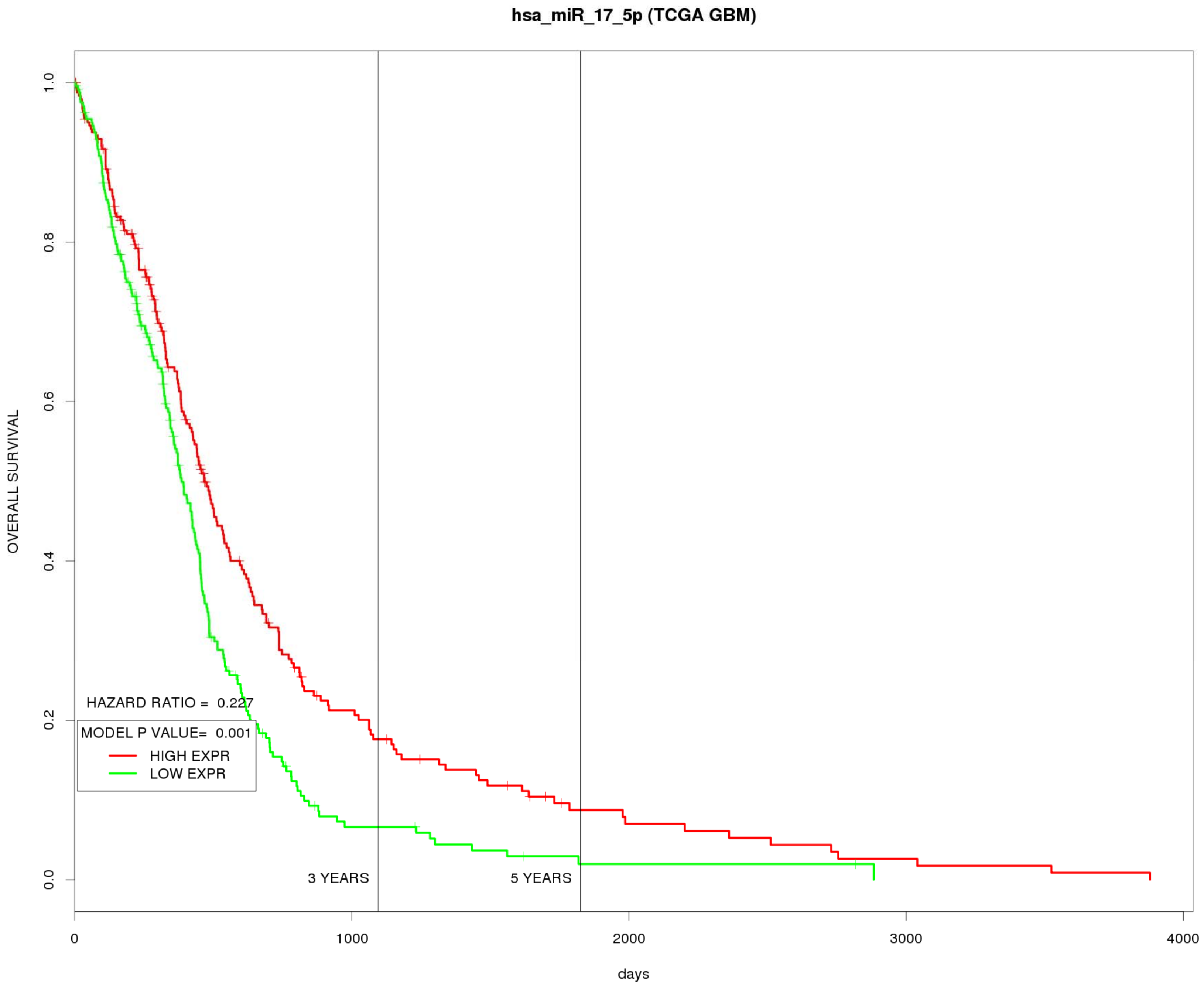

hsa_miR_17_5p (TCGA GBM)
OVERALL SURVIVAL
1.0
0.8
0.6
0.4
0.2
0.0
HAZARD RATIO = 0.227
MODEL P VALUE= 0.001
HIGH EXPR
LOW EXPR
3 YEARS
5 YEARS
0
1000
2000
3000
4000
days

Figure 10

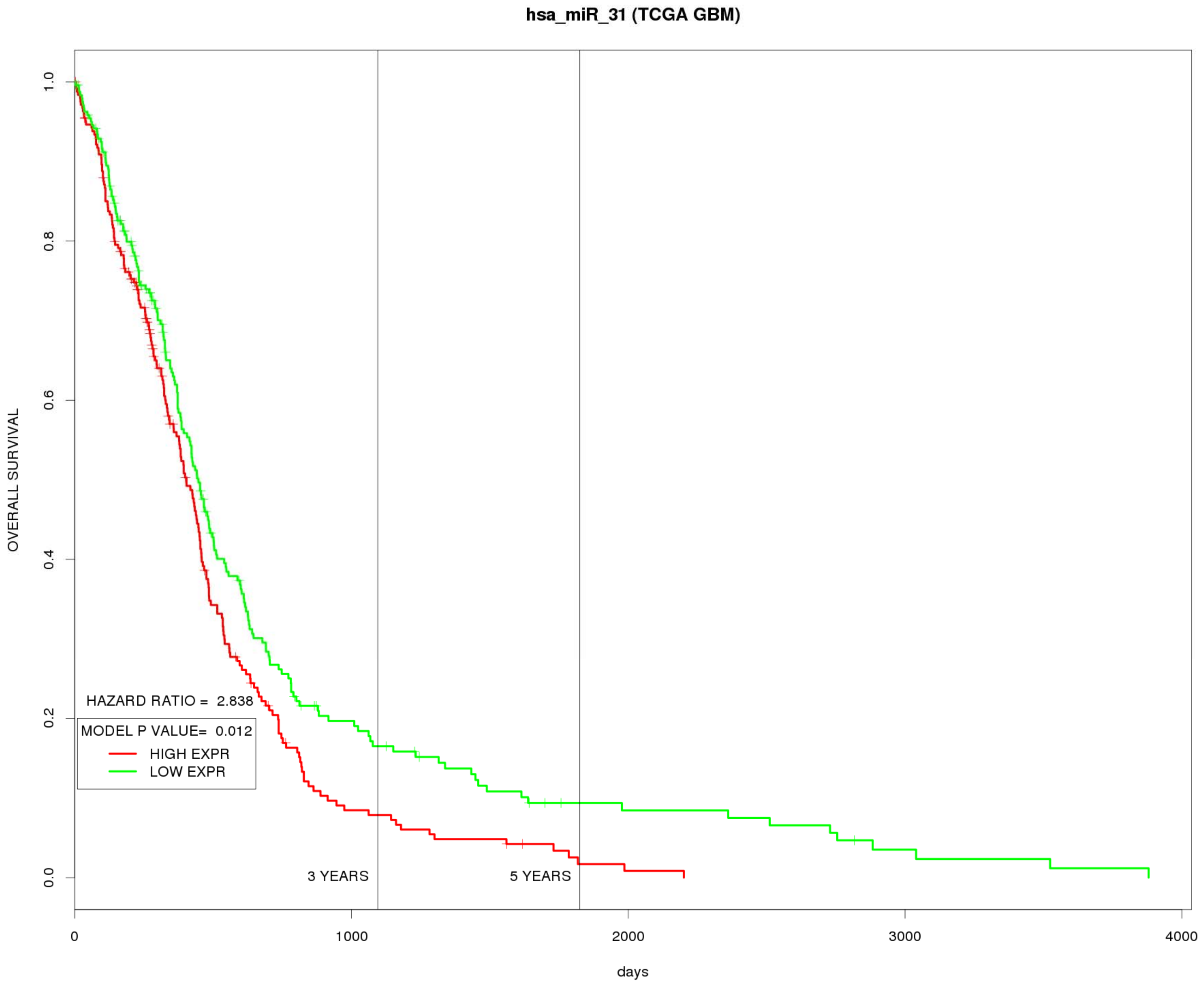

hsa_miR_31 (TCGA GBM)
OVERALL SURVIVAL
1.0
0.8
0.6
0.4
0.2
0.0
HAZARD RATIO = 2.838
MODEL P VALUE= 0.012
HIGH EXPR
LOW EXPR
3 YEARS
5 YEARS
0
1000
2000
3000
4000
days

Figure 11

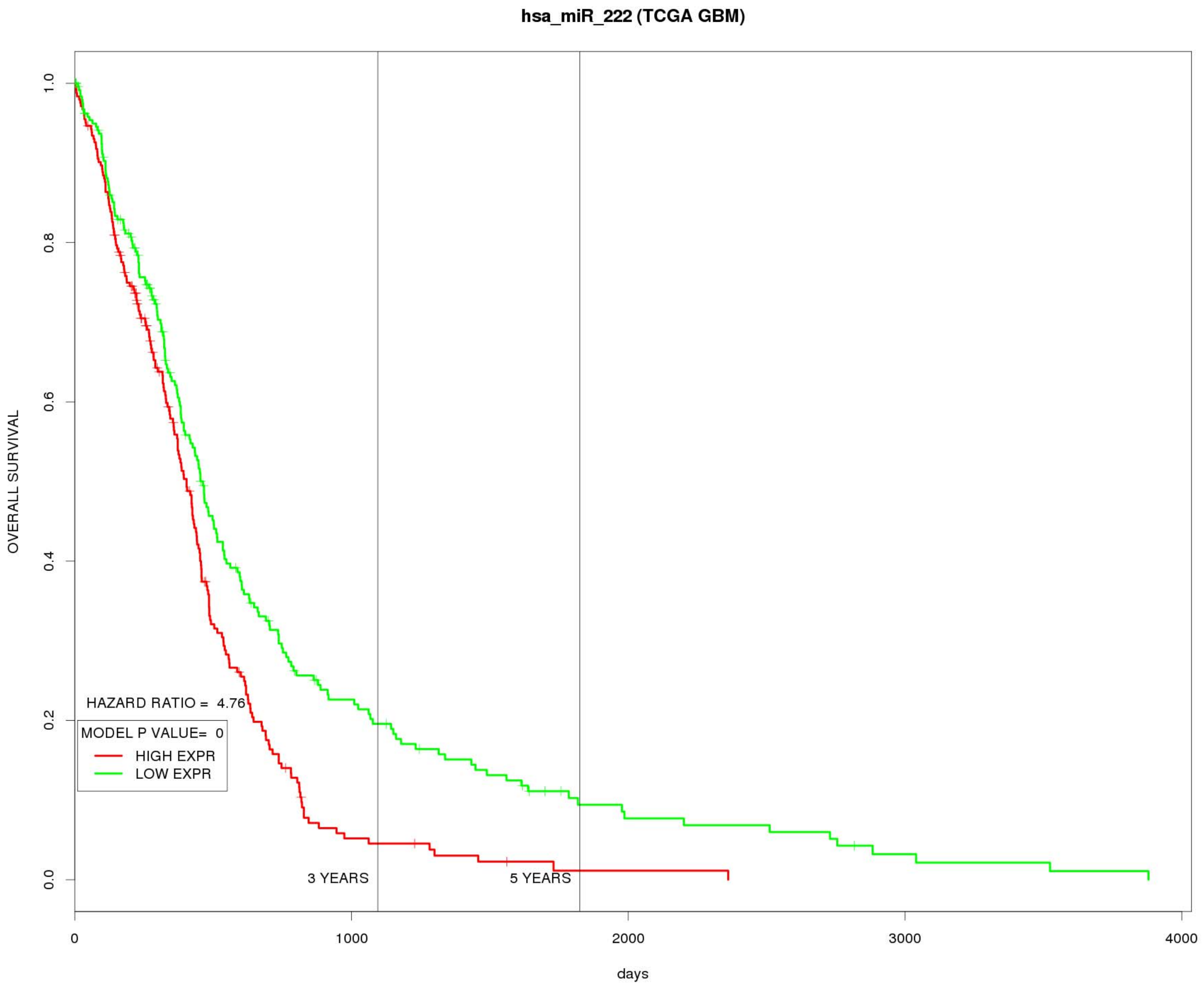

hsa_miR_222 (TCGA GBM)
OVERALL SURVIVAL
1.0
0.8
0.6
0.4
0.2
0.0
HAZARD RATIO = 4.76
MODEL P VALUE= 0
HIGH EXPR
LOW EXPR
3 YEARS
5 YEARS
0
1000
2000
3000
4000
days

Figure 12

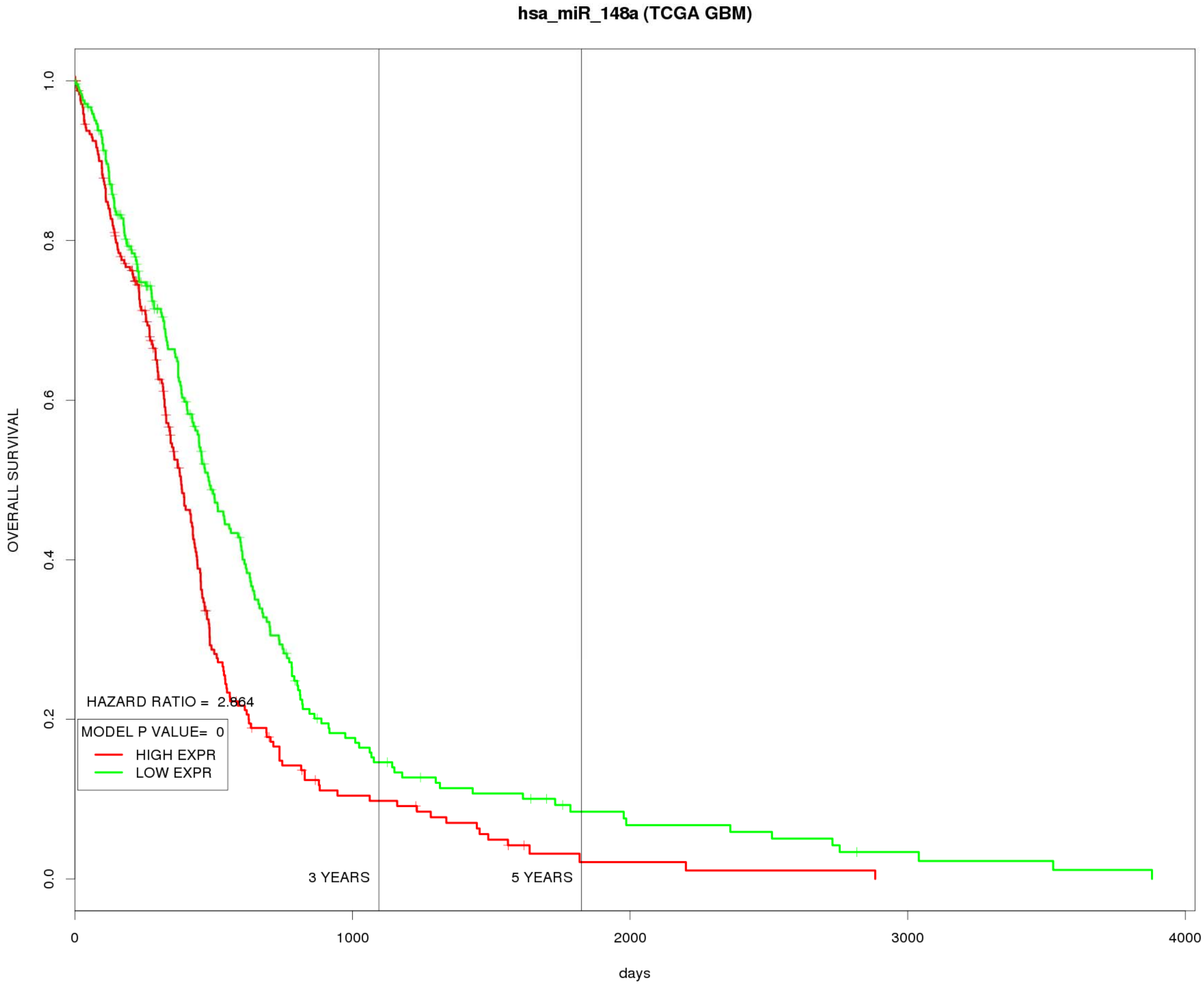

hsa_miR_148a (TCGA GBM)
OVERALL SURVIVAL
1.0
0.8
0.6
0.4
0.2
0.0
HAZARD RATIO = 2.864
MODEL P VALUE= 0
HIGH EXPR
LOW EXPR
3 YEARS
5 YEARS
0
1000
2000
3000
4000
days

Figure 13

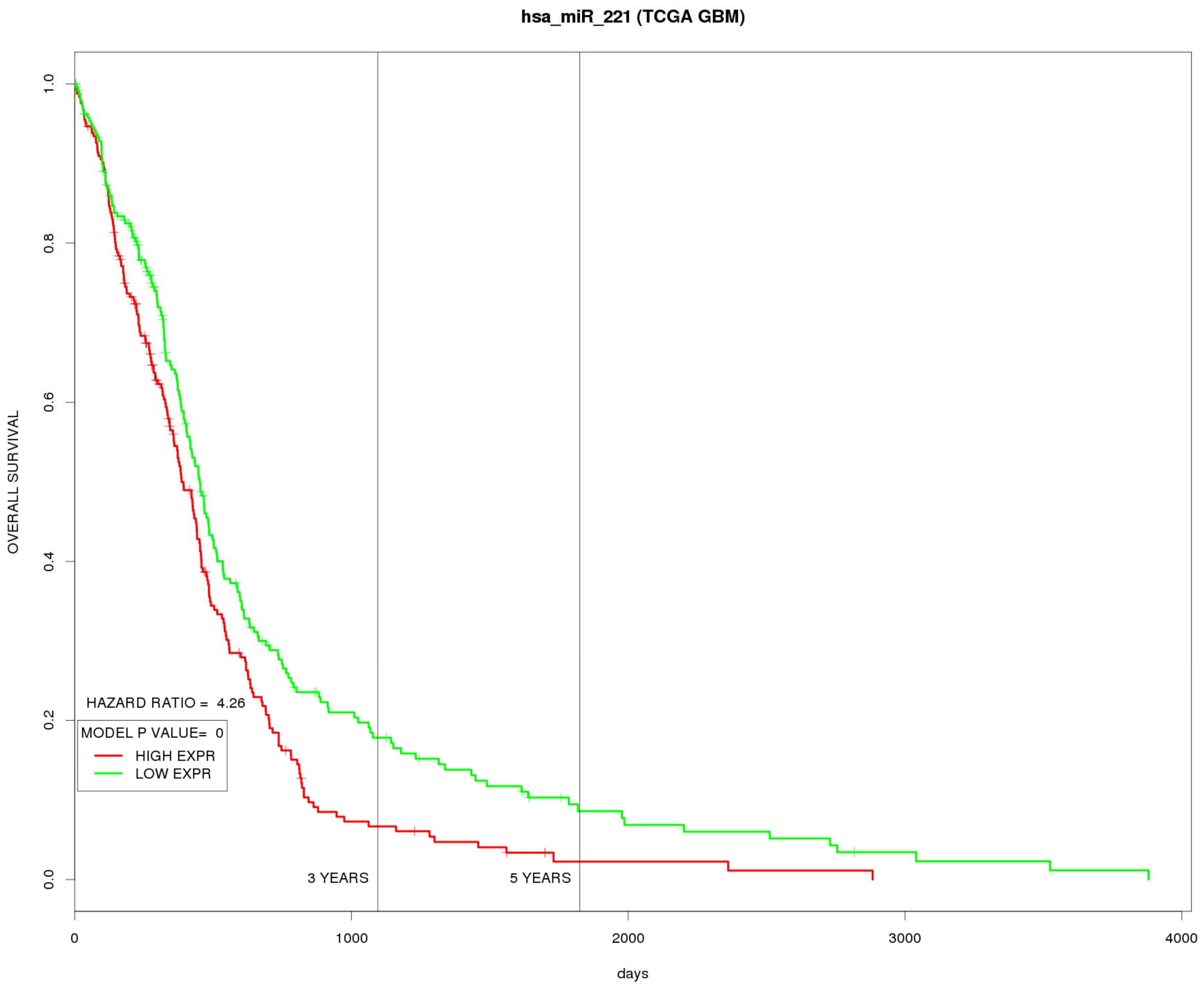

hsa_miR_221 (TCGA GBM)
OVERALL SURVIVAL
1.0
0.8
0.6
0.4
0.2
0.0
HAZARD RATIO = 4.26
MODEL P VALUE= 0
HIGH EXPR
LOW EXPR
3 YEARS
5 YEARS
0
1000
2000
3000
4000
days

Figure 14

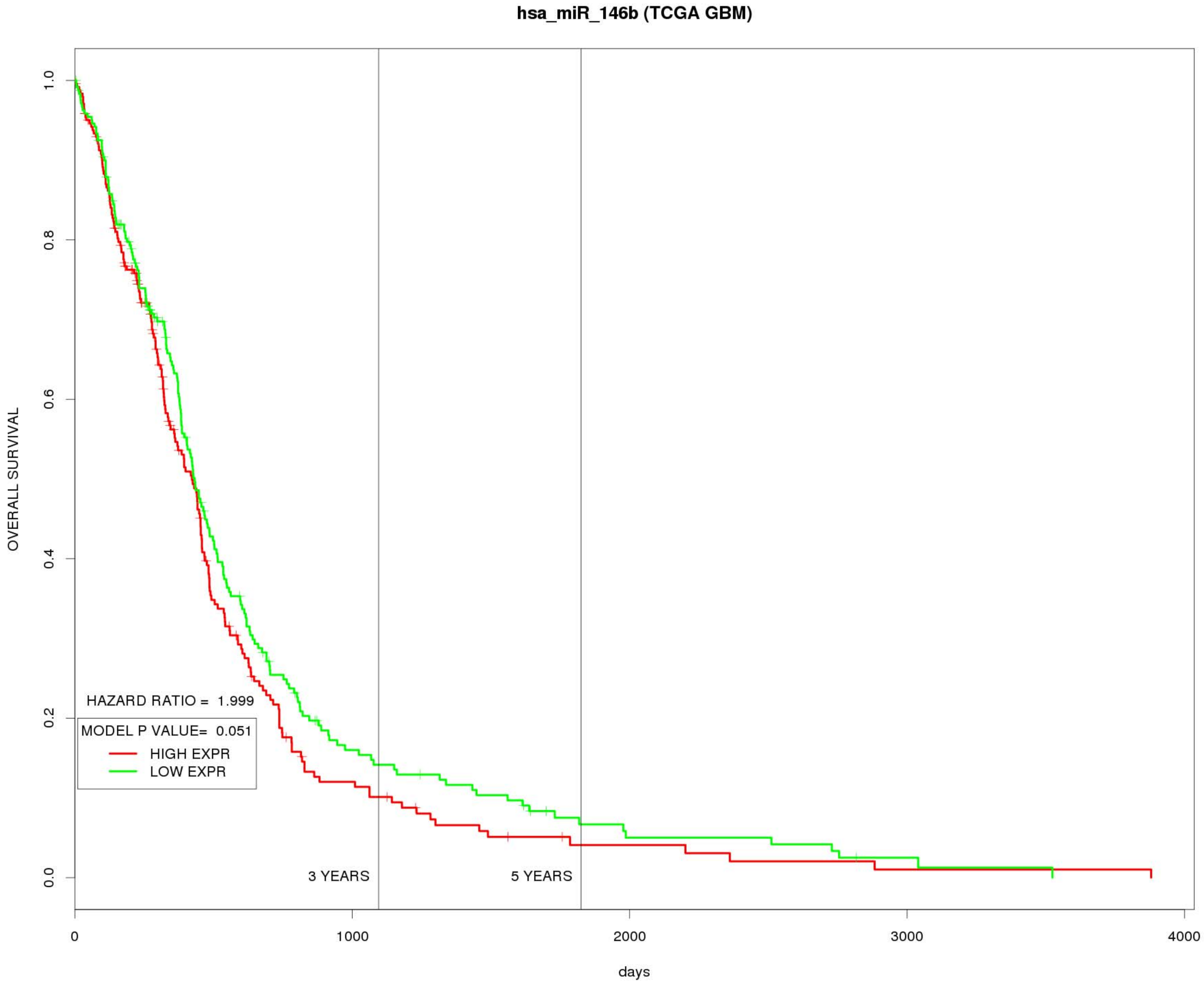
hsa_miR_146b (TCGA GBM)
OVERALL SURVIVAL
1.0
0.8
0.6
0.4
0.2
0.0
HAZARD RATIO = 1.999
MODEL P VALUE= 0.051
HIGH EXPR
LOW EXPR
3 YEARS
5 YEARS
0
1000
2000
3000
4000
days

Figure 15

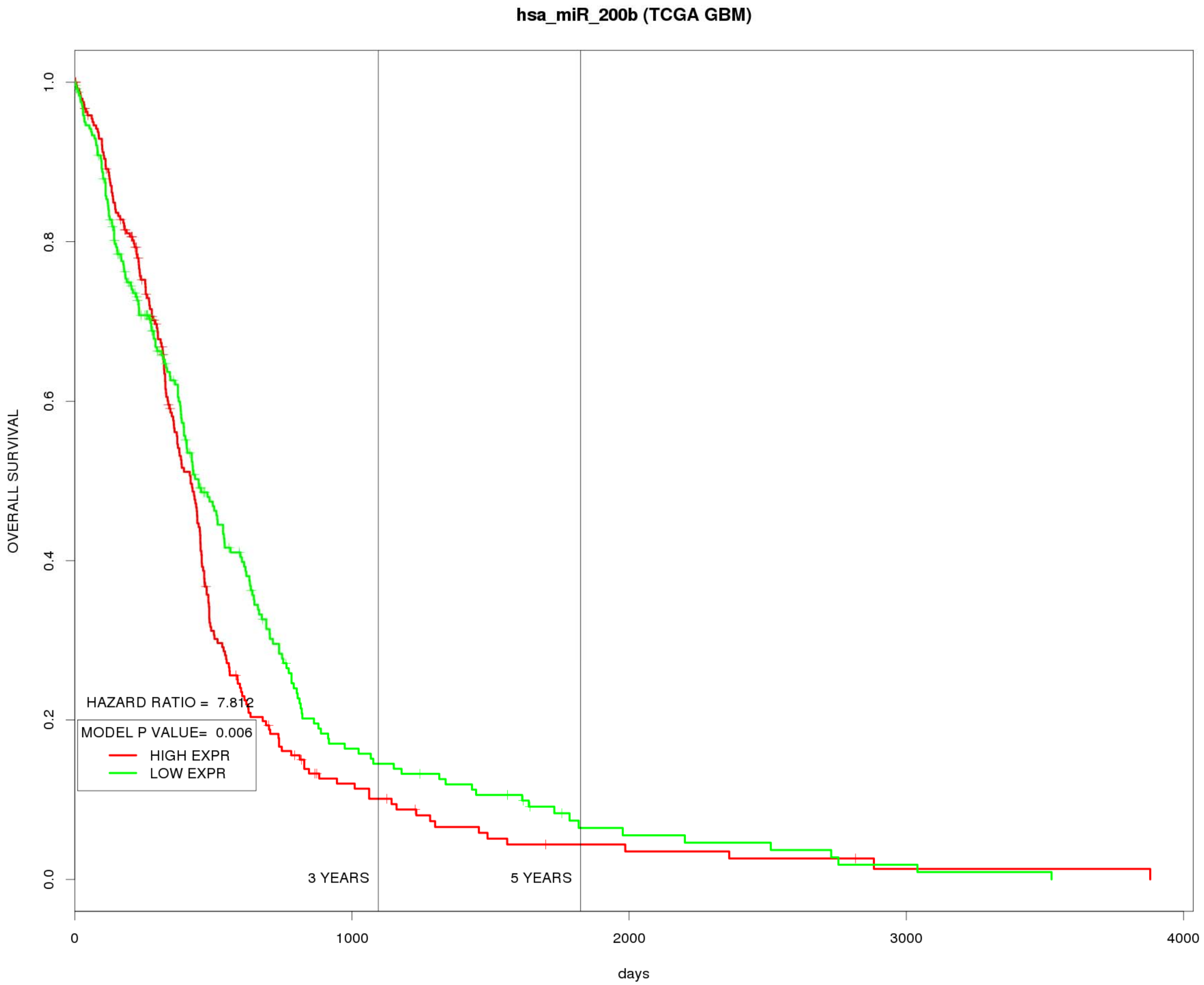

hsa_miR_200b (TCGA GBM)
OVERALL SURVIVAL
0.0
0.2
0.4
0.6
0.8
1.0
0
1000
2000
3000
4000
days
3 YEARS
5 YEARS
HAZARD RATIO = 7.812
MODEL P VALUE= 0.006
HIGH EXPR
LOW EXPR

Figure 16

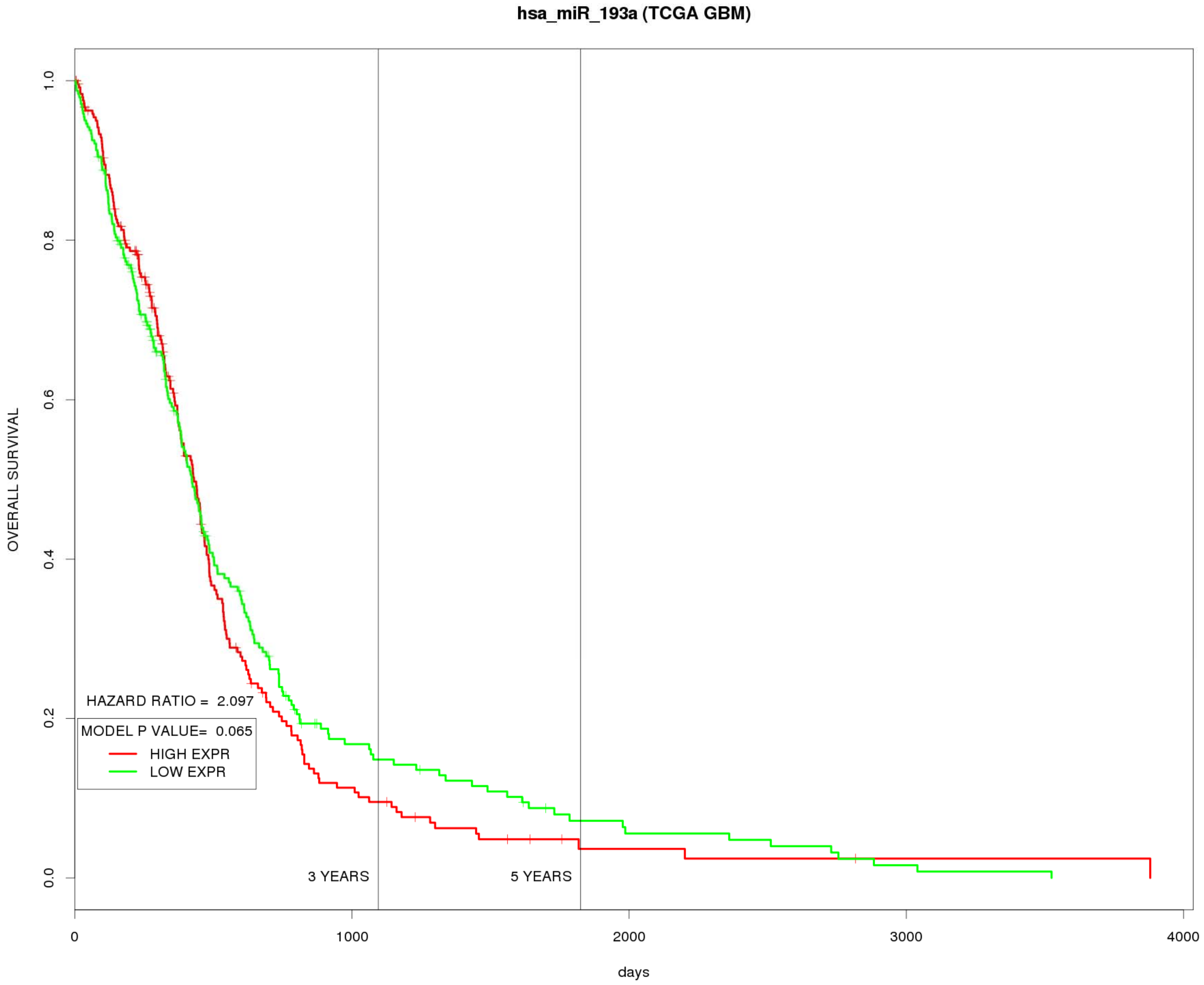

hsa_miR_193a (TCGA GBM)
OVERALL SURVIVAL
1.0
0.8
0.6
0.4
0.2
0.0
HAZARD RATIO = 2.097
MODEL P VALUE= 0.065
HIGH EXPR
LOW EXPR
3 YEARS
5 YEARS
0
1000
2000
3000
4000
days
